# Multi-utility of therapeutic drug monitoring in inflammatory bowel diseases

**DOI:** 10.3389/fmed.2022.864888

**Published:** 2022-07-28

**Authors:** Camilla de Almeida Martins, Karoline Soares Garcia, Natália Sousa Freita Queiroz

**Affiliations:** ^1^Department of Gastroenterology, University of São Paulo School of Medicine, São Paulo, Brazil; ^2^Hospital Santa Cruz, Curitiba, Brazil

**Keywords:** Crohn’s disease, ulcerative colitis, biologics, drug concentrations, therapeutic drug monitoring

## Abstract

Inflammatory bowel disease (IBD) treatment targets have progressed over time from clinical response to clinical and endoscopic remission. Several data have shown a positive correlation between serum biologic drug concentrations and favorable therapeutic outcomes. Therapeutic drug monitoring (TDM) has evolved as an important approach for optimizing the use of immunobiologics, especially antitumor necrosis factor therapy, in patients with IBD. The use of TDM is supported by medical societies and IBD experts in different contexts; however, challenges remain due to knowledge gaps that limit the widespread use of it. The aim of this review is to assess the role of TDM in IBD, focusing on the implementation of this strategy in different scenarios and demonstrating the multi-utility aspects of this approach in clinical practice.

## Introduction

Treatment goals for patients with inflammatory bowel disease (IBD) have evolved over time from clinical response to deep remission (clinical and endoscopic remission), aiming for a change in the disease course (1). Therapeutic drug monitoring (TDM), which involves measuring serum drug concentrations and anti-drug antibody (ADA) concentrations, has been recognized as a useful tool for biological therapy optimization along with early and scheduled disease assessment to ensure maintenance of remission in IBD (2).

Several studies have demonstrated an association between serum biologic drug concentrations and favorable therapeutic outcomes, while subtherapeutic drug concentrations and immunogenicity can explain a substantial proportion of treatment failure (2). A recent large prospective observational multicenter study from the United Kingdom, PANTS, which enrolled 1,610 biologic-naïve patients with Crohn’s disease (CD) treated with infliximab or adalimumab, demonstrated that treatment failure to infliximab and adalimumab is common and is predicted by low drug concentrations, mediated in part by immunogenicity (3). In multivariate analysis, drug concentration at week 14 was the major independent risk factor associated with time to immunogenicity for both drugs. In addition, clinical covariates, such as inflammatory burden, albumin levels, and patient-related factors, have been recognized as factors that can influence pharmacokinetic variability for all biologics (4). Even though these circumstances may reasonably justify the adoption of TDM routinely in clinical practice, there are still many barriers to the widespread use of TDM (5).

The use of TDM is supported by medical societies and IBD experts in different situations (1, 2, 6–13). In 2017, the American Gastroenterology Association (AGA) recommended the use of reactive TDM to help treatment decisions in patients with IBD with active disease who are being treated with anti-tumor necrosis factor (anti-TNF). They make no suggestions about the use of routine proactive TDM (14). The American College of Gastroenterology (ACG) published a recent literature review and expert consensus that has advised the use of TDM in a reactive context for all biologics and proactive TDM for anti-TNF as well as following a drug holiday or previously to treatment de-escalation (12). Table 1 summarizes recommendations regarding TDM of both guidelines. There are still many knowledge gaps in the literature, such as the most appropriate measurement timepoints, proper interpretation of the results, and the identification of the optimal thresholds to target.

**TABLE 1:** Summary of AGA and ACG guidelines.

AGA guideline (14)	Suggested trough level (μ g/mL)
Reactive TDM for anti-TNF treatment in active IBD	Infliximab > 5 Adalimumab ≥ 7.5 Certolizumab ≥ 20 Golimumab unknown
No recommendation about proactive TDM for anti-TNF treatment in quiescent IBD	

**ACG guideline (12)**	**Suggested trough level (μ g/mL)**

Reactive TDM for all biologics (primary non response and secondary loss of response)	Infliximab: At week 2: > 20–25 Week 6: > 15–20 Week 14: 7–10 Maintenance: 5–10 Adalimumab: Week 4: 8–12 Maintenance: 8–12
Proactive TDM for anti-TNF therapy (after induction, at least once in maintenance, treatment de-escalation, drug holiday, anti-TNF monotherapy)	

AGA, american gastroenterology association; ACG, american college of gastroenterology.

**FIGURE 1:**
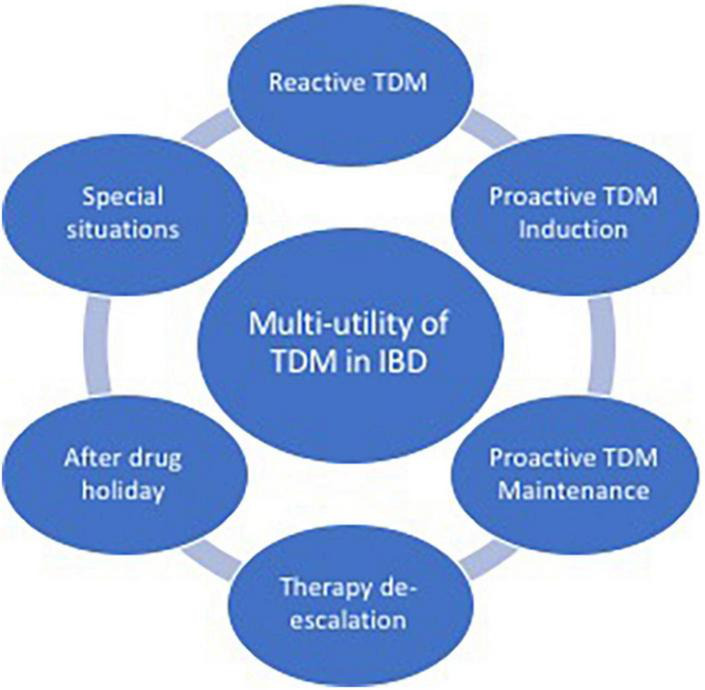
Aspects of multi-utility of therapeutic drug monitoring (TDM) in inflammatory bowel disease (IBD).

In this review, we aim to explore the role of TDM in IBD, focusing on the applicability of this strategy in different scenarios, and illustrating the multi-utility aspects of this approach in clinical practice (Figure 1).

## Proactive therapeutic drug monitoring

Proactive TDM is defined as the measurement of drug trough concentrations (measuring drug level just before the subsequent infusion) and ADA levels to optimize drug concentration at specific time points (i.e., induction, at the end of induction, or maintenance) (7, 8). It is performed to optimize therapy in order to improve response rates and likely prevent future flares and loss of response (LOR) (6). Moreover, some recent data suggest that proactive TDM could also improve the safety and cost-effectiveness of biologic therapy, by preventing undetectable or low drug levels (9, 10, 15–18).

Several exposure-outcome relationship data from prospective studies and *post hoc* analyses of randomized controlled trials (RCTs) have demonstrated that higher induction, postinduction, and maintenance anti-TNF drug levels are associated with more favorable outcomes, indicating that anti-TNF therapy may benefit from proactive TDM to guide dose optimization (9, 13, 19). Here, we explore the clinical scenarios where proactive TDM might be useful.

### Induction

The induction phase has emerged as an important period to proactively adjust the biological serum concentrations. This phase is characterized by a high inflammatory burden, increased drug clearance, and consequently a greater risk of inadequate drug exposure. Thus, early optimization of biological therapy could potentially prevent primary non-response (PNR) and immunogenicity, providing clinical and pharmacoeconomic benefits (19).

#### Exposure–outcome relationship during induction

The relationship between inadequate serum drug levels and PNR has been explored in numerous studies. In a cohort of 25 patients with IBD initiating treatment with infliximab, Bar-Yoseph et al. identified that lower infliximab trough levels and higher antibody to infliximab titers were predictive of PNR (20). Verstockt et al. also demonstrated that adalimumab trough concentrations <8.3 μg/ml at week 4 were associated with a higher risk of detection of ADA at week 12 (21).

Moreover, proactive TDM at induction has been associated with better therapeutic outcomes at the end of the induction and during the maintenance period compared with empiric dose optimization, both in CD and ulcerative colitis (UC) (22–27). Papamichael et al. retrospectively evaluated 101 patients with UC and found that infliximab trough levels ≥15 μg/ml at week 6 and ≥2.1 μg/ml at week 14 were independent factors associated with short-term mucosal healing (22). Similarly, a *post hoc* analysis of 484 patients with UC from the active ulcerative colitis trials (ACT 1/2) demonstrated that infliximab trough levels ≥18.6 μg/ml at week 2 and ≥10.6 μg/ml at week 6 were associated with endoscopic remission at week 8 (23).

A *post hoc* analysis from the CLASSIC I/II trials also identified a positive relationship between adalimumab trough concentrations and clinical remission at week 4 in patients with moderate to severely active CD (24). Additionally, Davidov et al. identified that the infliximab trough level of >9.2 μg/ml at week 2 was associated with a fistula response at week 14 (25). Conversely, a recent RCT, NOR-DRUM study, evaluating 411 patients with chronic immune-mediated inflammatory diseases initiating infliximab therapy failed to demonstrate improvement in clinical remission rates at week 30 in the group undergoing TDM during induction compared with those on clinically based dosing. The trial did not have statistical power to test hypotheses within the IBD subgroup (28).

Furthermore, pharmacokinetics data have demonstrated that there is great interindividual variability in drug concentration vs. time profiles in biological fluids, and drug concentrations at induction can fluctuate more than during maintenance treatment (26, 29). Different studies have also demonstrated that the main covariates influencing infliximab trough level are the presence of ADA, evidence of a high inflammatory burden [elevated C-reactive protein (CRP), low albumin, and great extension of the disease], concomitant corticosteroid use, and infliximab monotherapy (26, 29).

It is important to point out that most of the data supporting the strategy of TDM during induction come from anti-TNF agents. Preliminary data related to other biologic drugs (vedolizumab and ustekinumab) has been emerging; however, it is still not possible to make recommendations regarding TDM with these specific agents (19).

#### Thresholds to target during induction

Although many observational studies reinforce the benefits of proactive TDM during induction, the threshold drug trough levels, as well as the best moment to measure it, have not yet been established. The target drug trough levels may vary according to the disease phenotype and desired therapeutic outcomes. A recent expert consensus statement on TDM of biologics in IBD by Cheifetz et al. supports the clinical utility of TDM during the induction phase for patients treated with anti-TNF agents, aiming at infliximab trough levels of 20–25 μg/ml at week 2 and 15–20 μg/ml at week 6, and adalimumab trough levels of 8–12 μg/ml at week 4 (12). Papamichael et al. proposed a simplified algorithm for TDM during infliximab induction therapy in IBD. They proposed that in the presence of an adequate infliximab trough level at week 2 or 6, patients in clinical response should continue on infliximab standard dose during the maintenance phase, but patients that show no response should switch the drug. In the group with therapeutic infliximab trough level, there is no recommendation for measuring antibody to infliximab since ADA is more clinically relevant when there is no detectable drug level. In contrast, individuals with undetectable or subtherapeutic infliximab trough levels should be assessed according to ADA levels. In this group, in the absence of antibody to infliximab or the presence of low titers of it, therapy optimization should be considered (either escalating the dose, decreasing the interval between the infusions, or adding immunosuppressants), while, in the presence of high-titer antibody to infliximab, switching therapy should be considered (30).

Table 2 summarizes the most relevant studies regarding TDM in the induction phase.

**TABLE 2:** Summary of main TDM studies in induction phase.

Observational studies	IBD type; *N*	Drug	Drug level target (μ g/mL)	Time point	Therapeutic outcome
**Prospective**					
Ungar et al. (POETIC)	CD; *N* = 91	Adalimumab	>6.7	Week 2	Clinical remission by week 14
Verstockt et al. (21)	CD; *N* = 116	Adalimumab	<8.3	Week 4	Presence of antibodies to adalimumab by week 12
Clarkston et al.	CD; *N* = 72	Infliximab	≥26.7	Week 2	Clinical response at week 14
			≥15.9	Week 6	
Buhl et al.	CD and UC; *N* = 166	Infliximab	>22.9	Week 2	Clinical response at week 14
			>11.8	Week 6	
**Retrospective**					
Dreesen et al.	CD; *N* = 122	Infliximab	>23.1	Week 2	Endoscopic remission at week 12
			>10	Week 6	
Vande Casteele et al. (23)	UC; *N* = 484	Infliximab	≥18.6	Week 2	Endoscopic remission at week 8
			≥10.6	Week 6	
Adedokun et al.	UC; *N* = 728	Infliximab	>22	Week 6	Clinical response at week 8

### Maintenance

Many TDM studies are related to the maintenance phase of immunobiological therapy. A retrospective study by Perinbasekar et al. evaluating 127 patients with IBD treated with infliximab or adalimumab observed that clinical response rates at 60 days and 1 year were higher in the proactive group in comparison to the control group. The proactive group had higher rates of endoscopic response (31). Bernardo et al. retrospectively included 117 patients with IBD and found that the period to relapse was significantly longer in the drug monitoring group and there was a trend toward higher therapeutic failure in the clinical-based adjustment group (32).

A multicenter and retrospective cohort study evaluated 264 patients with IBD on infliximab maintenance therapy and found that the proactive group was associated with better clinical outcomes, such as greater drug durability, less need for IBD-related surgery or hospitalization, and a lower risk of antibodies to infliximab or serious infusion reactions. In this study, an infliximab level of 3.55 and 4.65 μg/ml were identified as the optimal cut-off values for treatment failure and IBD-related hospitalization, respectively (16). Moreover, Papamichael et al. evaluated 102 patients with IBD on infliximab maintenance therapy and compared long-term outcomes between patients who did proactive monitoring after reactive TDM with reactive testing only. This study demonstrated that the proactive group, in which more than 90% of patients had an infliximab trough concentration of >5 μg/ml, had a greater rate of treatment persistence and fewer IBD-related hospitalizations than the reactive testing group alone (10).

Another multicenter and retrospective study of 382 patients with IBD has shown that proactive TDM of adalimumab on maintenance therapy might be associated with a lower risk of treatment failure in comparison to the standard of care in patients with IBD. They found that an adalimumab serum level threshold of 11.7 μg/ml differentiates between patients with or without treatment failure (33). Also, Morita et al. have demonstrated that the cut-off value of the trough level for predicting mucosal healing was 2.7 μg/ml for infliximab and 10.3 μg/ml for adalimumab in patients with UC (34).

Recently, the aforementioned PANTS study reported that week 14 drug trough levels of 7 mg/L for infliximab and 12 mg/L for adalimumab were associated with clinical remission at both weeks 14 and 54 (3).

Therefore, both retrospective and prospective observational studies encourage the use of proactive TDM. Concerning RCT, two studies have been inconclusive, while three more recent ones indicate that proactive TDM could be associated with favorable outcomes.

The landmark TAXIT trial (the Trough Level Adapted Infliximab Treatment) did not achieve its primary endpoint, given that 69 vs. 66% of patients in the concentration vs. clinically based dosing groups achieved combined clinical and biochemical remission 1 year after optimization, respectively (*p* = 0.686). Even so, important secondary outcomes were observed in the proactive TDM group, such as lower frequency of undetectable drug levels, less antibody formation, and a lower chance of flares (17). Moreover, it was demonstrated that dose de-escalation did not affect disease activity and reduced drug costs by 28%.

A retrospective study from Pouillon et al. on the long-term outcomes of all 226 patients who completed the TAXIT maintenance phase reported that infliximab discontinuation happened earlier in patients treated in the clinically based dosing group than in patients treated in the proactive TDM group during a follow-up of 41 months. In addition, concentration-based dosing was associated with longer treatment responses, low surgical rates, and corticosteroid use (35).

Another prospective, double-blind, and randomized study evaluating 122 patients with CD, the TAILORIX trial, showed that there was no difference in corticosteroid-free clinical remission between an increasing dose of infliximab based on a combination of symptoms, biomarkers, and serum drug levels and an increasing dose based on symptoms alone, starting at week 14. There were important limitations concerning the study design that could explain the unexpected results. For instance, in the control group, 60% of dose escalations based on symptoms had normal biomarkers, whereas 53% of possible dose escalations based on symptoms in the interventional arm were avoided as biomarkers were not elevated. Moreover, a minority of patients were dose escalated based on trough concentration (36).

The PAILOT trial was a prospective and randomized controlled study conducted with 78 biologic-naive children with CD who were randomly assigned into proactive vs. reactive TDM groups following response to adalimumab induction. The authors found that the proactive dose adjustment of adalimumab was associated with a higher rate of corticosteroid-free clinical remission at all visits from weeks 8 to 72 when compared with the reactive group (37).

Strik et al. conducted the PRECISION trial, enrolling 80 patients with IBD in clinical remission treated with infliximab in the maintenance phase. They were randomized into two groups; one received infliximab dosing guided by a Bayesian pharmacokinetic model, targeting the infliximab trough level of >3 μg/ml, and the other received conventional treatment. After 1 year, the study demonstrated that a higher proportion of patients from the infliximab dosing model group were in sustained clinical remission compared to the control group. In addition, the TDM group had lower median FCP levels (38).

Recently, a Norwegian multicenter trial conducted with 458 patients with immune-mediated inflammatory diseases undergoing maintenance therapy with infliximab has demonstrated that the proportion of patients with sustained disease control over 52 weeks of follow-up was significantly higher in the proactive TDM group compared with the standard therapy group. The cost-effectiveness, as well as the superiority of this strategy as compared with the reactive approach, remains to be demonstrated (39).

Concerning TDM in biologics other than the anti-TNF mechanism, there are only a few studies evaluating the exposure-response relationship, reinforcing that higher vedolizumab and ustekinumab concentrations are associated with favorable outcomes (40–43). There is an expert agreement that more data are needed to support the use of proactive TDM for biologics other than anti-TNF therapies (12). Tables 3, 4 summarize RCTs and observational studies regarding TDM in the maintenance phase, respectively.

**TABLE 3:** Summary of RCTs assessing the role of TDM in IBD.

RCT	IBD type; *N*	Groups	Drug	Drug level target (μ g/mL)	Primary endpoint
Steenholdt et al. (57)	CD *N* = 69	Reactive TDM vs. standard care	Infliximab	≥0.5	Cost-effectiveness and Crohn’s disease activity index response after 12 weeks
Vande Casteele et al. (17) (TAXIT)	CD and UC *N* = 263	Proactive TDM vs. clinically based	Infliximab	>3	Clinical and biochemical remission at 1 year after the optimization phase
D’Haens et al. (36) (TAILORIX)	CD *N* = 122	Dose optimization based on clinical symptoms and biomarkers and/or proactive TDM vs. clinical symptoms alone	Infliximab	>3	Sustained corticosteroid-free clinical remission from weeks 22 to 54 with mucosal healing at week 54
Assa et al. (37) (PAILOT)	Pediatric CD *N* = 78	Proactive vs. reactive TDM	Adalimumab	≥5	Sustained corticosteroid-free clinical remission from weeks 8 to 72
Strik et al. (38) (PRECISION)	CD and UC *N* = 80	Proactive TDM based on pharmacokinetic dashboard vs. standard dosing	Infliximab	>3	Sustained clinical remission after 1 year
Syversen et al. (39) (NOR-DRUM)	Rheumatoid arthritis, spondyloarthritis, psoriatic arthritis, UC, CD, and psoriasis *N* = 458	Part A – proactive TDM in induction phase vs. standard therapy Part B – proactive TDM in maintenance phase vs. standard therapy	Infliximab	>20 at the second infusion >15 at the third infusion Maintenance IFX 3–8	Part A – clinical remission at week 30 Part B – sustained disease control without disease worsening during 52 weeks

**TABLE 4:** Summary of most relevant observational proactive TDM studies in maintenance phase.

Observational studies	IBD type; *N*	Drug	Drug level target (μ g/mL)	Time point	Therapeutic outcome
**Prospective**					
Kennedy et al. (3) (PANTS)	CD; *N* = 1610	Infliximab	≥7.0	Week 14	Clinical remission at week 14 and 54
		Adalimumab	≥12		
**Retrospective**					
Perinbasekar et al. (31)	CD and UC; *N* = 127	Infliximab	≥3	At least once in maintenance	Clinical response at 60 days, clinical response at 1 year, endoscopic response and persistence with anti-TNF at 1 year
		Adalimumab	≥5		
Bernardo et al. (32)	CD and UC; *N* = 117	Infliximab	3–7 in CD; 5–10 in UC	Every 6 months	Clinical remission at week 48
		Adalimumab	5–7 in CD; 7–9 in UC		
Papamichael et al. (30)	CD and UC; *N* = 264	Infliximab	5–10	Any frequency during maintenance phase	Treatment failure (IFX discontinuation due to LOR or serious adverse event or surgery)
Papamichael et al. (18)	CD and UC; *N* = 102	Infliximab	5–10	Median of 3 (range 1–7) proactive infliximab monitoring evaluations	Treatment failure and IBD-related surgery and hospitalization
Papamichael et al. (33)	CD and UC; *N* = 382	Adalimumab	>10	At least once	Treatment failure from the start of adalimumab until the end of follow-up (3 years)

### Guiding treatment de-escalation

Another important role for proactive TDM is to guide treatment de-escalation of biological therapy. A prospective study by Amiot et al. reported that in patients with IBD in clinical remission, TDM-based adjustment is predictive of LOR following infliximab dose reduction. The authors concluded that therapy de-escalation of infliximab in patients in clinical remission should be guided by TDM rather than according to symptoms and CRP (44). Recently, a retrospective observational single-center study of 96 patients with IBD in remission showed that TDM-based adjustment (with infliximab trough levels of more than 7 mg/L) was associated with a decreased risk of relapse when compared to clinically based de-escalation (45).

A real-world cohort from Petitcollin et al. with 91 patients with IBD in remission showed that TDM could be beneficial for follow-up of patients after infliximab de-escalation (46). Furthermore, a prospective observational study of 87 patients with IBD suggested that a cut-off adalimumab level of 12.2 mg/ml could be appropriate in guiding dose reduction (47). The recent expert consensus statement on TDM recommended that dose de-escalation should be considered for infliximab or adalimumab trough concentrations that are consistently higher than 10–15 mg/ml (12).

Correspondingly, proactive TDM should be considered after withdrawal of immunosuppressive therapy (48, 49). A study by Drobne et al. that evaluated patients with CD using infliximab in combination with immunosuppressants observed that detectable infliximab trough level at the time of immunomodulator removal is associated with long-term response (49).

## Reactive therapeutic drug monitoring

Reactive TDM should be performed in the context of active disease to elucidate the mechanism of primary or secondary loss-of-response (SLR) to immunobiological therapy. Thus, this approach helps to guide treatment decisions, such as dose optimization, combination therapy with an immunomodulator, or switch in or out of class (14, 50).

Whether reactive TDM compared to empiric care is associated with better outcomes remains controversial. However, there are intuitive benefits to using TDM to elucidate the mechanism underlying anti-TNF LOR, such as the avoidance of futile, and potentially hazardous, dose intensification in patients with high titer antidrug antibodies (50).

A retrospective observational cohort study by Kelly et al. showed that the reactive TDM approach is associated with higher post-adjustment clinical response and endoscopic remission compared to clinical decision-making alone (51). Yanai et al. demonstrated that at the time of SLR, infliximab and adalimumab trough concentrations of more than 3.8 and 4.5 mg/ml, respectively, identified patients who benefited more from a switch to another mechanism than to dose escalation or switching to another antitumor necrosis factor (52).

Similarly, an interesting prospective study by Roblin et al. showed that, in patients with IBD presenting secondary LOR to adalimumab, low drug trough levels without antibodies are strongly predictive of clinical response in 67% of cases after adalimumab optimization. In addition, adalimumab trough concentrations of >4.9 μg/ml were associated with the failure of two anti-TNF agents (adalimumab and infliximab) in 90% of cases, and switching to another drug class should be cogitated (53).

Given that there are still limited treatment options for IBD, especially for certain phenotypes such as perianal fistulizing CD, the optimization of the first biologic is usually recommended as it typically results in a higher rate of efficacy when compared to subsequent biologic therapies (54, 55). Thus, the most recent expert consensus on TDM suggests that treatment discontinuation should not be considered until a trough level of at least 10–15 μg/ml is achieved for both infliximab and adalimumab therapies (12).

A recognizable unmet need when performing reactive TDM is the proper interpretation of ADA, as titers are often expressed in arbitrary units and cannot be directly compared between different assays (2). As such, to avoid the inappropriate withdrawal of a biologic due to hypothetical high-titer ADA, it is crucial to differentiate levels that can be overcome by treatment optimization (dose escalation, dose interval shortening, and/or addition of an immunomodulator) from high-titer ADA that can lead to undetectable or low drug concentrations, infusion reactions, and treatment failure (12). Although the specific cut-off identifying high-titer ADA remains uncertain for each assay, experts agree that low-titer antibodies to infliximab can be defined as 10 U/ml for the homogeneous mobility shift assay (12).

**FIGURE 2:**
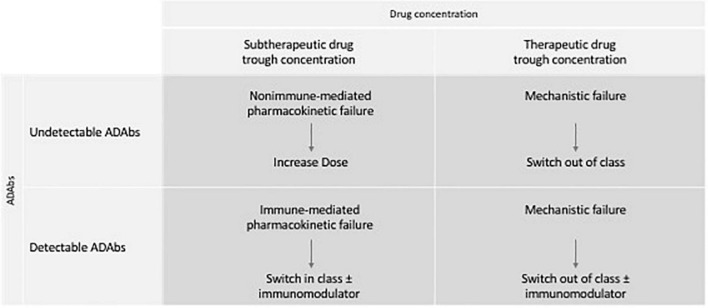
Approach of secondary loss of response of reactive TDM.

Besides guiding better therapeutic management, some studies have suggested that TDM-based dosing is less costly and more effective than empiric dose escalation in the setting of secondary LOR (56). Moreover, an RCT by Steenholdt et al. reported that reactive TDM was associated with important cost savings at 12 and 20 weeks and 1 year (57, 58). Therefore, most gastroenterology societies and expert groups recommend the use of reactive TDM for both PNR and secondary LOR (6, 12, 14). Figure 2 summarizes the approach to secondary LOR when TDM is available. Table 5 summarizes the most relevant observational studies regarding reactive TDM.

**TABLE 5:** Summary of most relevant reactive TDM studies.

Observational studies	IBD type; *N*	Drug	Drug level target (μ g/mL)	Time point	Therapeutic outcome
**Prospective**					
Guidi et al.	CD and UC; *N* = 148	Infliximab	>3	Loss of response including active endoscopic disease	Clinical outcomes 12 weeks after the therapeutic intervention
**Retrospective**					
Yanai et al. (52)	CD and UC	Infliximab	>3.8	Loss of response	Clinical efficacy of each intervention strategy instituted for loss of response
		Adalimumab	>4.5		
Kelly et al. (51)	CD and UC; *N* = 271	Infliximab	>4.5	Loss of response	Endoscopic remission 6 months after readjustment

## Therapeutic drug monitoring in special situations

### Following a drug holiday

In patients who have already experienced the LOR to a biologic agent, reexposure to the same drug is associated with a high risk of failure to treatment. In this specific scenario, TDM has been recognized as a promising strategy to optimize drug levels and avoid pharmacokinetic failure due to inadequate drug exposure (12, 59).

Assuming drug holiday as a delay (intentional or not) of at least 3 doses of a biological agent, an expert panel study published by Melmed et al. considers appropriate checking drug and ADA after the first reinduction dose (59). The ACG consensus also endorses proactive TDM after a long drug holiday as an approach to efficiently guide treatment decisions, and it recommends that TDM should be performed in patients restarting treatment with infliximab before the second dose. As there is no sufficient evidence, the authors made no statement regarding drug holidays with other biologic agents (12).

In a retrospective study by Baert et al. that evaluated 128 patients with IBD who restarted infliximab after a median 15-month discontinuation, the absence of antibody to infliximab before the second infusion and reinitiation therapy with concomitant immunomodulator were associated with the clinical response at weeks 10–14. This study also showed that the early detection of antibodies to infliximab (before second or third doses) after reexposure to infliximab was associated with higher rates of infusion reactions. For preventing severe infusion reactions, the authors suggest concomitant immunomodulator therapy (azathioprine/6-mercaptopurine or methotrexate) when reinitiating infliximab after a drug holiday, and it may also be reasonable not to administer subsequent doses if there is evidence of circulating ADA after the first reinduction dose (60).

### Perioperative care

Despite significant improvements in the medical management of IBD, surgery is still needed in a significant subset of patients during the course of the disease (61–63). Given that most patients who undergo surgery have been previously treated with biologics (64), the proper understanding of the impact of serum drug concentrations on perioperative outcomes is paramount. However, data regarding serum concentrations of biologics in the perioperative period are still conflicting.

A retrospective Canadian study by Waterman et al. analyzed the results of 473 CD-related surgical procedures (195 in patients under previous anti-TNFs and 278 in matched controls) (65). No significant differences were observed in the length of stay, rates of urinary tract infection, pneumonia, bacteremia, readmission, reoperation, or mortality between groups. The authors also showed that detectable infliximab levels did not increase the rates of postoperative wound infection (*p* = 0.21).

A prospective study by Lau et al. evaluating 123 patients with CD undergoing abdominal surgery demonstrated that infliximab concentration above 3 μg/ml was associated with an increased rate of overall complications (OR 2.5; *p* = 0.03) and infectious complications (OR 3.0; *p* = 0.03) (66). The increase in overall complications and readmission rates was more significant in patients with drug concentrations above 8 μg/ml. Conversely, no difference was observed in postoperative morbidity in patients with UC with undetectable concentrations [31/77 (40%)] and patients with detectable infliximab concentrations [8/17 (41%)], *p* = 0.61.

The largest prospective multicenter trial assessing the risk of surgery and biologics (*The Postoperative Infection in Inflammatory Bowel Disease*—PUCCINI) was presented at Digestive Disease Week (DDW) 2019 (67). Among a total of 955 procedures (382 with the use of anti-TNFs up to 12 weeks before surgery), the rates of overall infectious complications did not differ between patients with previous exposure to anti-TNFs and controls (20 vs. 19.4%, *p* = 0.801) or detectable serum anti-TNF concentrations (19.7 vs. 19.6%, *p* = 0.985). Accordingly, no differences in the rates of surgical site infections were found in patients with exposure to anti-TNFs (12.4 vs. 11.5%, *p* = 0.692) or detectable drug concentrations (10.3 vs. 12.1%, *p* = 0.513).

There is only one study assessing the effect of preoperative vedolizumab drug concentrations on postoperative outcomes in patients with IBD undergoing major abdominal surgery (68). Among 72 patients with IBD (42 UC and 27 CD), no differences in postoperative morbidity were observed between patients with detectable (>1.6 mcg/ml) and undetectable vedolizumab concentrations. Likewise, there is just a single report assessing the impact of preoperative ustekinumab concentrations on postoperative surgical outcomes in 36 patients with IBD (31 CD, 4 UC, and 1 IBD-unclassified). Ustekinumab concentrations were detectable (≥0.9 μg/ml) in 25 (69%) and undetectable in 11 (31%) patients (69). There were no significant differences between groups regarding overall postoperative morbidity (27 vs. 28%, *p* = 0.72), 30-day readmission rate (18 vs. 8%, *p* = 0.57), postoperative ileus (18 vs. 8%, *p* = 0.57), or wound infection (9 vs. 4%, *p* = 0.52).

### Perianal fistulizing Crohn’s disease

The perianal fistulizing CD comprises a disabling phenotype of IBD whose clinical course may tremendously affect patients’ quality of life. Studies have demonstrated that higher serum concentrations of anti-TNF agents are associated with higher rates of fistula closure. A *post hoc* analysis of ACCENT-II showed that infliximab trough concentrations at week 14 were associated with fistula response at weeks 14 and 54 (70). Higher concentrations of infliximab at week 14 were independently associated with both fistula response and normalization of CRP at week 14 (OR: 2.32; 95% CI: 1.55–3.49; *p* < 0.001). Infliximab trough levels predictive of fistula response and CRP normalization at week 14 were ≥20.2 μg/ml at week 2, ≥15 μg/ml at week 6, and ≥7.2 μg/ml at week 14.

Early induction infliximab levels were also associated with perianal fistula response. A retrospective observational study evaluating 36 patients with perianal fistulas demonstrated that infliximab drug levels of 9.25 μg/ml at week 2 and 7.25 μg/ml at week 6 were the best predictors of cessation or significant improvement of fistula drainage (25). Moreover, a cross-sectional study that included 117 patients with CD with perianal fistula found that levels of infliximab ≥10 μg/ml were also associated with higher fistula healing rates (71).

### Acute severe ulcerative colitis

Despite the introduction of salvage therapies such as cyclosporine and infliximab, management of acute severe UC remains challenging and colectomy is still required in a subset of refractory patients (72, 73). Failure to infliximab treatment has been associated with low drug exposure as a consequence of increased inflammatory burden, high drug clearance, and fecal loss (74–77).

Emerging data support that the achievement of higher drug levels during induction correlates with endoscopic remission for UC. In a *post hoc* analysis from the ACT 1 and 2 trials including 484 patients with UC, infliximab levels of ≥18.6 μg/ml at week 2 and ≥10.6 μg/ml at week 6 were associated with endoscopic remission at week 8 (23).

A recent retrospective study by Battat et al. showed that higher clearance of infliximab and, consequently, lower serum concentrations are associated with a greater chance of colectomy in 39 patients with acute severe UC. The median baseline calculated clearance of infliximab was higher in patients with colectomy at 6 months than in patients without (0.733 vs. 0.569 L/day; *p* = 0.005) (76). A clearance threshold of infliximab of 0.627 L/day identified patients who required colectomy with 80.0% sensitivity and 82.8% specificity (AUC, 0.80). In addition, the multivariable analysis identified that the baseline infliximab clearance value was the only factor associated with colectomy.

Based on the current data, emphasis should be given to studying the role of TDM in acute severe UC and choosing the optimal infliximab dosing aiming for improvements in clinical outcomes.

## Conclusion

Therapeutic drug monitoring is supported by both retrospective and prospective studies, and this approach has progressively evolved as the standard of care for patients with IBD on any biologics. Although there is some conflicting data, proactive TDM is beneficial for improving outcomes for patients with IBD on anti-TNFs. Patients with a higher risk of increased clearance and immunogenicity are more likely to benefit from proactive drug monitoring. Future prospective studies assessing the role of TDM in special situations are eagerly awaited.

## Author contributions

CM, KG, and NQ wrote the manuscript. All authors critically reviewed the content of the manuscript and approved the submission of the manuscript.
